# Crystal structure of {2-methyl-2-[(pyridin-2-yl­meth­yl)amino]­propan-1-ol-κ^3^ *N*,*N*′,*O*}bis­(nitrato-κ*O*)copper(II) from synchrotron data

**DOI:** 10.1107/S2056989018018352

**Published:** 2019-01-04

**Authors:** Jong Won Shin, Dae-Woong Kim, Jae-Woo Jeon, Dohyun Moon

**Affiliations:** a Daegu-Gyeongbuk Branch, Korea Institute of Science and Technology Information, 10 Exco-ro, Buk-gu, Daegu 41515, Republic of Korea; b Beamline Department, Pohang Accelerator Laboratory, 80 Jigokro-127-beongil, Nam-Gu Pohang, Gyeongbuk 37673, Republic of Korea; c Department of Electronic Materials Science and Engineering, Kyungpook National University, Daegu 41566, Republic of Korea; d SEGI RETECH, 1-67 Ogyegongdan-gil, Yeongcheon, Gyeongbuk, 38882, Republic of Korea

**Keywords:** crystal structure, π–π inter­action, hydrogen bond, synchrotron data

## Abstract

The title compound, [Cu(NO_3_)_2_(C_10_H_16_N_2_O)], has been synthesized and characterized by synchrotron single-crystal diffraction at 100 K. The Cu^II^ ion has a distorted square-pyramidal coordination geometry with two N and one O atoms of the C_10_H_16_N_2_O ligand and one nitrate anion in the equatorial plane and another nitrate anion at the axial position. The equatorial Cu—N and Cu—O bond lengths are in the range 1.9608 (14)–2.0861 (15) Å, which are shorter than the axial Cu—O_nitrate_ bond length [2.1259 (16) Å]. In the crystal, mol­ecules are linked *via* inter­molecular N—H⋯O and O—H⋯O hydrogen bonds, forming a sheet structure parallel to the *bc* plane. The sheets are further linked through a face-to-face π–π inter­action [centroid–centroid distance = 3.994 (1) Å]. Weak inter­molecular C—H⋯O inter­actions are also observed in the sheet and between adjacent sheets.

## Abstract 2

The Cu^II^ ion in the title compound shows a distorted square-pyramidal coordination geometry. In the crystal, the mol­ecules are connected by N—H⋯O, O—H⋯O, C—H⋯O and π–π inter­actions, forming a three-dimensional supra­molecular network.

## Chemical context   

Transition-metal complexes containing amine or its derivative ligands have attracted considerable attention owing to their diverse coordination geometries and their various applications in catalysis (Ahn *et al.*, 2017 ▸), as magnetic mat­erials (Liu, Zhou *et al.*, 2017 ▸) and fluorescent substances (Chia & Tay, 2014 ▸) as well as sensing materials (Liu, Wang *et al.*, 2017 ▸). In addition, polyamine ligands containing hydroxyl groups can easily form multinuclear complexes (such as dinuclear or trinuclear) with various transition-metal ions and hydrogen-bonded supra­molecular compounds due to the deprotonation of hydroxyl groups by the transition-metal ions and anions (Shin *et al.*, 2014 ▸). For example, *N*-(2-pyridyl­meth­yl)iminodi­ethanol and *N*-(2-pyridyl­meth­yl)imino­diiso­propanol ligands containing amine, pyridine and hydroxyl groups have been used to form trinuclear metal complexes with cobalt and nickel ions, respectively, and these complexes have shown significant olefin epoxidations and magnetic inter­actions (Shin, Jeong *et al.*, 2016 ▸). The nitrate anion is a good candidate for the construction of multinuclear complexes or supra­molecular compounds by bridging metal ions or hydrogen bonding adjacent mol­ecules (El-Khatib *et al.*, 2018 ▸). Here, we report the preparation and crystal structure of a copper(II) complex, [Cu(C_10_H_16_N_2_O)(NO_3_)_2_], formed with a functional tridentate ligand, 2-methyl-2-[(2-pyridinylmeth­yl)amino]-1-propanol, and nitrate anions.
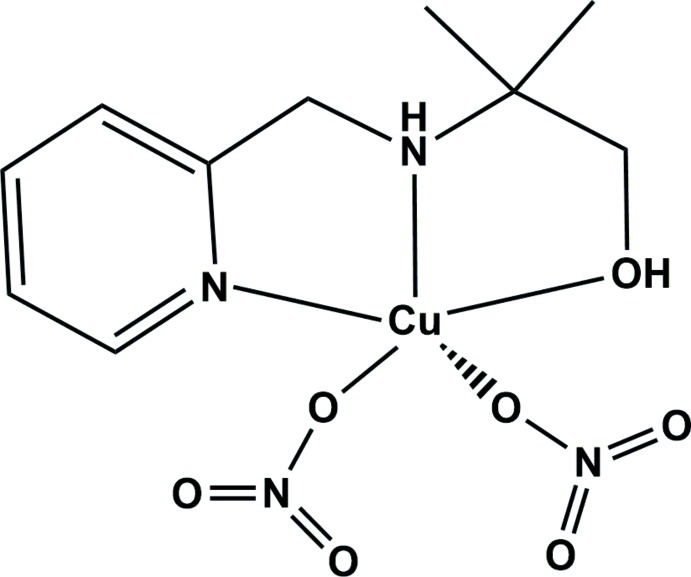


## Structural commentary   

**Figure 1:**
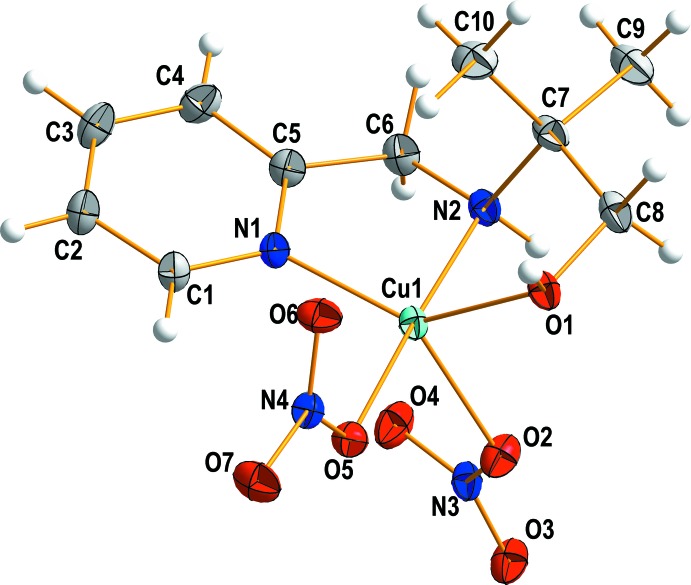
View of the mol­ecular structure of the title compound, showing the atom-labelling scheme, with displacement ellipsoids drawn at the 50% probability.

A view of the mol­ecular structure of the title compound is shown in Fig. 1 ▸. The central Cu^II^ ion is coordinated by two nitro­gen and one oxygen atoms from the C_10_H_16_N_2_O ligand and by two oxygen atoms from nitrate anions, and adopts a distorted square-pyramidal geometry. The equatorial plane consists of the two nitro­gen atoms (N1 and N2) and the oxygen atom (O1) of the hydroxyl group in the C_10_H_16_N_2_O ligand and one oxygen atom (O5) of the nitrate anion. The coordination geometry is completed by an oxygen atom (O2) from the other nitrate anion in the axial position. The equatorial bond lengths, Cu—N and Cu—O, are in the range 1.9608 (14) to 2.0861 (15) Å. The axial bond length, Cu—O_nitrate_, is 2.1259 (16) Å. The average length of the Cu—N and Cu—O bonds between the Cu^II^ ion and the C_10_H_16_N_2_O ligand is 2.0081 (8) Å, which is shorter than the average bond length in the reported [Cu(C_10_H_16_N_2_O)Cl_2_] complex possessing the same ligand and metal (Shin, Lee *et al.*, 2016 ▸). The axial bond length is also shorter than that in [Cu(C_10_H_16_N_2_O)Cl_2_], which can be attributed to the size effect of the coordinated anions. The nitrate anions are coordinated in a *cis* position to each other and the axial bond is longer than the equatorial bond. The bite angles N1—Cu1—N2 and N2—Cu1—O1 in the five-membered chelate rings are 84.53 (7) and 82.92 (7)°, respect­ively.

## Supra­molecular features   

**Figure 2:**
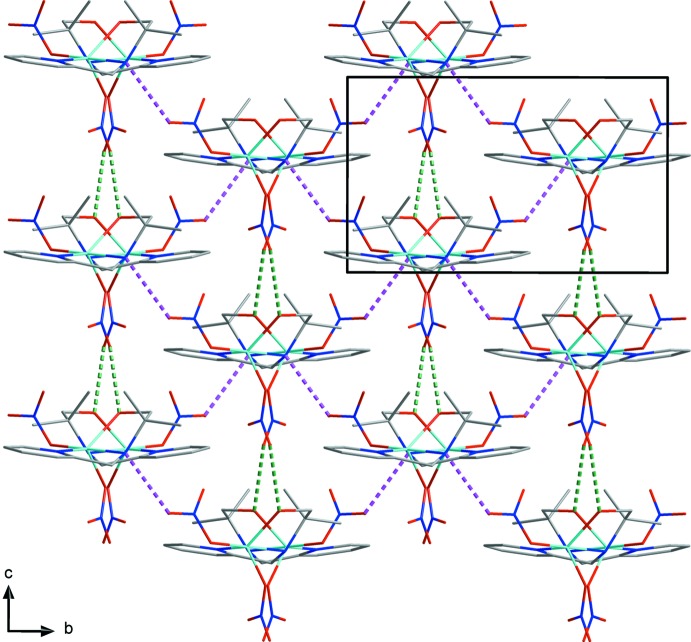
A packing diagram of the title compound viewed along the *a* axis, showing the N—H⋯O (purple dashed lines) and O—H⋯O (dark green dashed lines) hydrogen bonds.

**Figure 3:**
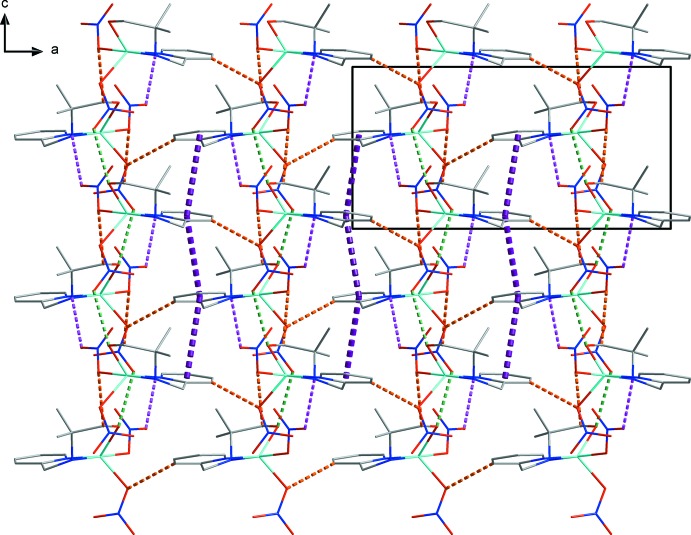
A packing diagram of the title compound viewed along the *b* axis, showing the N—H⋯O (purple dashed lines), O—H⋯O (dark green dashed lines) and C—H⋯O (orange dashed lines) hydrogen bonds as well as π–π inter­actions (violet dashed lines).

In the crystal, two nitrate anions form inter­molecular hydrogen bonds (O1—H1*O*1⋯O3^i^, N2—H2⋯O7^ii^ and C8—H8*AB*⋯O5^iv^; symmetry codes as in Table 1 ▸) with adjacent C_10_H_16_N_2_O ligands, generating an undulating sheet structure parallel to the *bc* plane (Fig. 2 ▸). Another C—H⋯O hydrogen bond (C4—H4⋯O2^iii^; Table 1 ▸) links the sheets into a three-dimensional structure (Fig. 3 ▸). Moreover, the sheets are linked by a π–π inter­action between pyridine rings; the distance between the centroids of the pyridine rings is 3.994 (1) Å and the dihedral angle is 19.317 (1)°.

## Database survey   

A search of the Cambridge Structural Database (Version 5.39, update of August 2018; Groom *et al.*, 2016 ▸) shows only one mononuclear copper(II) complex with the same C_10_H_16_N_2_O ligand, for which the synthesis and crystal structure have been reported (Shin, Lee *et al.*, 2016 ▸). A similar copper(II) complex with poly(2,6-dimethyl-1,4-phenyl­ene ether) ligands involving secondary amine, pyridine and hydroxyl groups has been prepared to study its catalytic activities (Guieu *et al.*, 2004 ▸).

## Synthesis and crystallization   

The C_10_H_16_N_2_O ligand was prepared according to a slight modification of the previous reported method (Shin, Lee *et al.*, 2016 ▸). To a methanol solution (10 mL) of Cu(NO_3_)_2_·3H_2_O (200 mg, 0.823 mmol) was added dropwise a methanol solution (10 mL) of C_10_H_16_N_2_O (149 mg, 0.823 mmol); the colour became dark blue, and the solution was stirred for 30 min at room temperature. Blue crystals of the title compound were obtained by diffusion of diethyl ether into the dark-blue solution for several days, and collected by filtration and washed with diethyl ether and dried in air (yield: 189 mg, 62%). FT–IR(ATR, cm^−1^): 3215, 3168, 3071, 2967, 1607, 1506, 1384, 1278, 1065, 1020.

## Refinement   

Crystal data, data collection and structure refinement details are summarized in Table 2 ▸. C-bound H atoms were placed in geometrically idealized positions and constrained to ride on their parent atoms, with C—H = 0.95–0.99 Å, and with *U*_iso_(H) = 1.5 or 1.2*U*_eq_(C). The positions of the O- and N-bound H atoms were assigned based on a difference-Fourier map, and were refined with distance restraints of O—H = 0.84 (1) Å and N—H = 1.00 (1) Å, and with *U*_iso_(H) = 1.5*U*_eq_(O) and 1.2*U*_eq_(N). One reflection with a poor agreement between the measured and calculated intensities was omitted from the final refinement cycles.

## Supplementary Material

Crystal structure: contains datablock(s) I. DOI: 10.1107/S2056989018018352/is5504sup1.cif

Structure factors: contains datablock(s) I. DOI: 10.1107/S2056989018018352/is5504Isup3.hkl

CCDC reference: 1887447

Additional supporting information:  crystallographic information; 3D view; checkCIF report

## Figures and Tables

**Table 1:** Hydrogen-bond geometry (Å, °)

*D*—H⋯*A*	*D*—H	H⋯*A*	*D*⋯*A*	*D*—H⋯*A*
O1—H1*O*1⋯O3^i^	0.83 (1)	1.89 (2)	2.685 (2)	159 (4)
N2—H2⋯O7^ii^	0.98 (1)	1.97 (2)	2.930 (3)	167 (3)
C4—H4⋯O2^iii^	0.95	2.31	3.204 (3)	156
C8—H8*AB*⋯O5^iv^	0.99	2.55	3.455 (3)	152

Symmetry codes: (i) 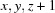; (ii) 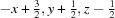; (iii) 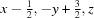; (iv) 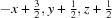.

**Table 2:** Experimental details

Crystal data	
Chemical formula	[Cu(NO_3_)_2_(C_10_H_16_N_2_O)]
*M*_r_	367.81
Crystal system, space group	Orthorhombic, *P* *n* *a*2_1_
Temperature (K)	100
*a*, *b*, *c* (Å)	14.990 (3), 12.520 (3), 7.6290 (15)
*V* (Å^3^)	1431.8 (5)
*Z*	4
Radiation type	Synchrotron, λ = 0.630 Å
μ (mm^−1^)	1.13
Crystal size (mm)	0.11 × 0.10 × 0.08

Data collection	
Diffractometer	ADSC Q210 CCD area detector
Absorption correction	Empirical (using intensity measurements) (*HKL3000sm *SCALEPACK**; Otwinowski & Minor, 1997 ▸)
*T*_min_, *T*_max_	0.912, 1.000
No. of measured, independent and observed [*I* > 2σ(*I*)] reflections	13671, 4431, 4234
*R*_int_	0.032
(sin θ/λ)_max_ (Å^−1^)	0.720

Refinement	
*R*[*F* ^2^ > 2σ(*F* ^2^)], *wR*(*F* ^2^), *S*	0.025, 0.066, 1.08
No. of reflections	4431
No. of parameters	208
No. of restraints	3
H-atom treatment	H atoms treated by a mixture of independent and constrained refinement
Δρ_max_, Δρ_min_ (e Å^−3^)	0.40, −0.68
Absolute structure	Flack *x* determined using 1883 quotients [(*I* ^+^)−(*I* ^−^)]/[(*I* ^+^)+(*I* ^−^)] (Parsons *et al.*, 2013 ▸)
Absolute structure parameter	0.037 (5)

Computer programs: *PAL BL2D-SMDC* (Shin, Eom *et al.*, 2016 ▸), *HKL3000sm* (Otwinowski & Minor, 1997 ▸), *SHELXT2014* (Sheldrick, 2015*a* ▸), *SHELXL2018* (Sheldrick, 2015*b* ▸), *DIAMOND* (Putz & Brandenburg, 2014 ▸) and *publCIF* (Westrip, 2010 ▸).

## supplementary crystallographic information

### Crystal data

**Table:**

[Cu(NO_3_)_2_(C_10_H_16_N_2_O)]	*D*_x_ = 1.706 Mg m^−^^3^
*M**_r_* = 367.81	Synchrotron radiation, λ = 0.630 Å
Orthorhombic, *P**n**a*2_1_	Cell parameters from 29915 reflections
*a* = 14.990 (3) Å	θ = 0.4–33.6°
*b* = 12.520 (3) Å	µ = 1.13 mm^−^^1^
*c* = 7.6290 (15) Å	*T* = 100 K
*V* = 1431.8 (5) Å^3^	Block, blue
*Z* = 4	0.11 × 0.10 × 0.08 mm
*F*(000) = 756	

### Data collection

**Table:**

ADSC Q210 CCD area detector diffractometer	4234 reflections with *I* > 2σ(*I*)
Radiation source: PLSII 2D bending magnet	*R*_int_ = 0.032
ω scan	θ_max_ = 27.0°, θ_min_ = 1.9°
Absorption correction: empirical (using intensity measurements) (*HKL3000sm SCALEPACK*; Otwinowski & Minor, 1997)	*h* = −21→21
*T*_min_ = 0.912, *T*_max_ = 1.000	*k* = −18→18
13671 measured reflections	*l* = −10→10
4431 independent reflections	

### Refinement

**Table:**

Refinement on *F*^2^	H atoms treated by a mixture of independent and constrained refinement
Least-squares matrix: full	*w* = 1/[σ^2^(*F*_o_^2^) + (0.0434*P*)^2^ + 0.0982*P*] where *P* = (*F*_o_^2^ + 2*F*_c_^2^)/3
*R*[*F*^2^ > 2σ(*F*^2^)] = 0.025	(Δ/σ)_max_ = 0.002
*wR*(*F*^2^) = 0.066	Δρ_max_ = 0.40 e Å^−^^3^
*S* = 1.08	Δρ_min_ = −0.68 e Å^−^^3^
4431 reflections	Extinction correction: *SHELXL2018* (Sheldrick, 2015*b*), Fc^*^=kFc[1+0.001xFc^2^λ^3^/sin(2θ)]^-1/4^
208 parameters	Extinction coefficient: 0.026 (3)
3 restraints	Absolute structure: Flack *x* determined using 1883 quotients [(*I*^+^)-(*I*^-^)]/[(*I*^+^)+(*I*^-^)] (Parsons *et al.*, 2013)
Hydrogen site location: mixed	Absolute structure parameter: 0.037 (5)

### Special details

**Table:**

Geometry. All esds (except the esd in the dihedral angle between two l.s. planes) are estimated using the full covariance matrix. The cell esds are taken into account individually in the estimation of esds in distances, angles and torsion angles; correlations between esds in cell parameters are only used when they are defined by crystal symmetry. An approximate (isotropic) treatment of cell esds is used for estimating esds involving l.s. planes.

### Fractional atomic coordinates and isotropic or equivalent isotropic displacement parameters (Å^2^)

**Table:**

	*x*	*y*	*z*	*U*_iso_*/*U*_eq_	
Cu1	0.70732 (2)	0.68798 (2)	0.59459 (4)	0.01603 (8)	
O1	0.76542 (12)	0.78901 (12)	0.77964 (19)	0.0201 (3)	
H1O1	0.773 (2)	0.766 (3)	0.881 (2)	0.030*	
O2	0.79419 (10)	0.74637 (16)	0.3963 (2)	0.0245 (3)	
O3	0.81254 (12)	0.75969 (13)	0.1158 (2)	0.0264 (3)	
O4	0.68899 (14)	0.69393 (15)	0.2203 (3)	0.0301 (4)	
O5	0.79776 (9)	0.57547 (12)	0.6143 (3)	0.0201 (3)	
O6	0.75455 (11)	0.55182 (13)	0.8859 (2)	0.0256 (3)	
O7	0.85090 (12)	0.44393 (14)	0.7666 (2)	0.0291 (3)	
N1	0.60049 (10)	0.59461 (12)	0.5810 (3)	0.0173 (3)	
N2	0.61927 (12)	0.80764 (12)	0.5732 (3)	0.0188 (3)	
H2	0.638 (2)	0.856 (2)	0.479 (3)	0.023*	
N3	0.76463 (13)	0.73260 (14)	0.2413 (2)	0.0193 (3)	
N4	0.80075 (11)	0.52272 (14)	0.7611 (3)	0.0181 (3)	
C1	0.59808 (12)	0.48835 (14)	0.6052 (3)	0.0202 (3)	
H1	0.652533	0.451412	0.624769	0.024*	
C2	0.51962 (13)	0.43077 (14)	0.6027 (4)	0.0236 (3)	
H2A	0.520110	0.355445	0.617692	0.028*	
C3	0.43973 (14)	0.48534 (17)	0.5779 (3)	0.0267 (4)	
H3	0.384490	0.448145	0.578571	0.032*	
C4	0.44214 (14)	0.59522 (18)	0.5522 (3)	0.0251 (4)	
H4	0.388472	0.634150	0.534687	0.030*	
C5	0.52366 (14)	0.64731 (16)	0.5523 (2)	0.0196 (4)	
C6	0.53236 (15)	0.76513 (16)	0.5116 (3)	0.0223 (4)	
H6A	0.527092	0.776224	0.383523	0.027*	
H6AB	0.483274	0.804667	0.569281	0.027*	
C7	0.61826 (15)	0.87026 (16)	0.7416 (3)	0.0213 (4)	
C8	0.71738 (16)	0.88814 (17)	0.7830 (3)	0.0241 (4)	
H8A	0.743334	0.937761	0.695808	0.029*	
H8AB	0.723273	0.921226	0.900327	0.029*	
C9	0.57393 (18)	0.97932 (19)	0.7166 (3)	0.0307 (5)	
H9A	0.603838	1.017915	0.621562	0.046*	
H9AB	0.578676	1.020589	0.825375	0.046*	
H9AC	0.510874	0.969297	0.686921	0.046*	
C10	0.57262 (18)	0.80604 (18)	0.8857 (3)	0.0278 (5)	
H10A	0.511114	0.790176	0.850852	0.042*	
H10B	0.572316	0.847605	0.994588	0.042*	
H10C	0.605093	0.739069	0.904426	0.042*	

### Atomic displacement parameters (Å^2^)

**Table:**

	*U*^11^	*U*^22^	*U*^33^	*U*^12^	*U*^13^	*U*^23^
Cu1	0.01865 (12)	0.01345 (11)	0.01599 (12)	−0.00077 (6)	−0.00037 (10)	0.00057 (10)
O1	0.0285 (8)	0.0172 (6)	0.0146 (6)	−0.0006 (6)	−0.0019 (6)	−0.0012 (5)
O2	0.0284 (8)	0.0316 (8)	0.0136 (7)	−0.0090 (6)	−0.0019 (5)	0.0027 (6)
O3	0.0362 (8)	0.0279 (7)	0.0152 (7)	−0.0099 (6)	0.0022 (7)	0.0028 (6)
O4	0.0301 (9)	0.0375 (10)	0.0226 (8)	−0.0144 (7)	−0.0013 (7)	−0.0045 (6)
O5	0.0222 (6)	0.0196 (6)	0.0184 (8)	0.0025 (4)	0.0013 (6)	0.0035 (6)
O6	0.0281 (8)	0.0301 (7)	0.0185 (7)	0.0043 (6)	0.0045 (6)	0.0005 (6)
O7	0.0355 (9)	0.0255 (7)	0.0263 (7)	0.0120 (6)	0.0004 (7)	0.0058 (6)
N1	0.0183 (6)	0.0162 (6)	0.0174 (7)	−0.0012 (5)	−0.0016 (7)	−0.0004 (7)
N2	0.0242 (7)	0.0156 (6)	0.0167 (9)	0.0008 (5)	0.0001 (7)	0.0010 (6)
N3	0.0264 (8)	0.0160 (7)	0.0154 (7)	−0.0037 (6)	−0.0004 (7)	0.0005 (5)
N4	0.0187 (7)	0.0186 (8)	0.0171 (7)	−0.0013 (5)	−0.0021 (6)	0.0006 (6)
C1	0.0208 (7)	0.0161 (7)	0.0238 (8)	−0.0016 (5)	−0.0048 (9)	0.0012 (8)
C2	0.0247 (8)	0.0204 (7)	0.0256 (8)	−0.0056 (6)	−0.0042 (10)	0.0022 (9)
C3	0.0217 (8)	0.0299 (9)	0.0284 (11)	−0.0072 (7)	−0.0047 (9)	0.0051 (9)
C4	0.0176 (8)	0.0298 (9)	0.0279 (11)	0.0003 (7)	−0.0041 (7)	0.0049 (7)
C5	0.0207 (9)	0.0202 (8)	0.0179 (8)	0.0011 (6)	−0.0026 (6)	0.0028 (6)
C6	0.0247 (10)	0.0205 (9)	0.0217 (9)	0.0023 (7)	−0.0043 (7)	0.0040 (7)
C7	0.0296 (10)	0.0179 (8)	0.0165 (8)	0.0038 (7)	0.0034 (8)	−0.0002 (6)
C8	0.0334 (11)	0.0174 (9)	0.0215 (10)	0.0000 (7)	−0.0005 (8)	−0.0042 (7)
C9	0.0414 (13)	0.0215 (9)	0.0292 (10)	0.0091 (9)	0.0044 (10)	−0.0016 (8)
C10	0.0348 (12)	0.0297 (11)	0.0188 (9)	0.0028 (8)	0.0057 (9)	0.0029 (7)

### Geometric parameters (Å, º)

**Table:**

Cu1—O5	1.9608 (14)	C2—C3	1.392 (3)
Cu1—N1	1.9854 (16)	C2—H2A	0.9500
Cu1—N2	2.0033 (17)	C3—C4	1.390 (3)
Cu1—O1	2.0861 (15)	C3—H3	0.9500
Cu1—O2	2.1259 (16)	C4—C5	1.385 (3)
O1—C8	1.435 (3)	C4—H4	0.9500
O1—H1O1	0.834 (13)	C5—C6	1.513 (3)
O2—N3	1.274 (2)	C6—H6A	0.9900
O3—N3	1.244 (2)	C6—H6AB	0.9900
O4—N3	1.243 (3)	C7—C10	1.524 (3)
O5—N4	1.301 (3)	C7—C9	1.531 (3)
O6—N4	1.232 (2)	C7—C8	1.535 (3)
O7—N4	1.241 (2)	C8—H8A	0.9900
N1—C1	1.344 (2)	C8—H8AB	0.9900
N1—C5	1.345 (2)	C9—H9A	0.9800
N2—C6	1.484 (3)	C9—H9AB	0.9800
N2—C7	1.505 (3)	C9—H9AC	0.9800
N2—H2	0.977 (13)	C10—H10A	0.9800
C1—C2	1.380 (2)	C10—H10B	0.9800
C1—H1	0.9500	C10—H10C	0.9800
			
O5—Cu1—N1	97.97 (6)	C2—C3—H3	120.6
O5—Cu1—N2	177.48 (6)	C5—C4—C3	119.3 (2)
N1—Cu1—N2	84.53 (7)	C5—C4—H4	120.4
O5—Cu1—O1	95.45 (7)	C3—C4—H4	120.4
N1—Cu1—O1	136.81 (7)	N1—C5—C4	121.63 (18)
N2—Cu1—O1	82.92 (7)	N1—C5—C6	115.97 (17)
O5—Cu1—O2	83.01 (7)	C4—C5—C6	122.34 (18)
N1—Cu1—O2	131.25 (7)	N2—C6—C5	111.13 (16)
N2—Cu1—O2	95.08 (8)	N2—C6—H6A	109.4
O1—Cu1—O2	91.00 (6)	C5—C6—H6A	109.4
C8—O1—Cu1	109.09 (13)	N2—C6—H6AB	109.4
C8—O1—H1O1	111 (3)	C5—C6—H6AB	109.4
Cu1—O1—H1O1	118 (3)	H6A—C6—H6AB	108.0
N3—O2—Cu1	113.62 (12)	N2—C7—C10	110.21 (17)
N4—O5—Cu1	117.05 (13)	N2—C7—C9	111.27 (18)
C1—N1—C5	119.01 (16)	C10—C7—C9	111.44 (19)
C1—N1—Cu1	126.70 (13)	N2—C7—C8	104.00 (16)
C5—N1—Cu1	114.22 (13)	C10—C7—C8	111.27 (19)
C6—N2—C7	116.65 (17)	C9—C7—C8	108.41 (19)
C6—N2—Cu1	109.64 (12)	O1—C8—C7	110.84 (17)
C7—N2—Cu1	109.06 (13)	O1—C8—H8A	109.5
C6—N2—H2	103.6 (18)	C7—C8—H8A	109.5
C7—N2—H2	108.1 (19)	O1—C8—H8AB	109.5
Cu1—N2—H2	109.5 (18)	C7—C8—H8AB	109.5
O4—N3—O3	122.21 (18)	H8A—C8—H8AB	108.1
O4—N3—O2	119.33 (18)	C7—C9—H9A	109.5
O3—N3—O2	118.45 (17)	C7—C9—H9AB	109.5
O6—N4—O7	123.36 (19)	H9A—C9—H9AB	109.5
O6—N4—O5	119.70 (17)	C7—C9—H9AC	109.5
O7—N4—O5	116.95 (18)	H9A—C9—H9AC	109.5
N1—C1—C2	122.58 (17)	H9AB—C9—H9AC	109.5
N1—C1—H1	118.7	C7—C10—H10A	109.5
C2—C1—H1	118.7	C7—C10—H10B	109.5
C1—C2—C3	118.62 (17)	H10A—C10—H10B	109.5
C1—C2—H2A	120.7	C7—C10—H10C	109.5
C3—C2—H2A	120.7	H10A—C10—H10C	109.5
C4—C3—C2	118.86 (18)	H10B—C10—H10C	109.5
C4—C3—H3	120.6		
			
Cu1—O2—N3—O4	4.0 (3)	C7—N2—C6—C5	−101.79 (19)
Cu1—O2—N3—O3	−176.76 (14)	Cu1—N2—C6—C5	22.8 (2)
Cu1—O5—N4—O6	−7.5 (2)	N1—C5—C6—N2	−20.6 (2)
Cu1—O5—N4—O7	172.10 (14)	C4—C5—C6—N2	162.32 (19)
C5—N1—C1—C2	−0.4 (4)	C6—N2—C7—C10	52.6 (2)
Cu1—N1—C1—C2	176.29 (18)	Cu1—N2—C7—C10	−72.26 (19)
N1—C1—C2—C3	−1.4 (4)	C6—N2—C7—C9	−71.6 (2)
C1—C2—C3—C4	1.6 (4)	Cu1—N2—C7—C9	163.60 (15)
C2—C3—C4—C5	−0.1 (4)	C6—N2—C7—C8	171.93 (16)
C1—N1—C5—C4	1.9 (3)	Cu1—N2—C7—C8	47.09 (17)
Cu1—N1—C5—C4	−175.13 (15)	Cu1—O1—C8—C7	32.1 (2)
C1—N1—C5—C6	−175.2 (2)	N2—C7—C8—O1	−52.1 (2)
Cu1—N1—C5—C6	7.7 (2)	C10—C7—C8—O1	66.5 (2)
C3—C4—C5—N1	−1.7 (3)	C9—C7—C8—O1	−170.60 (18)
C3—C4—C5—C6	175.3 (2)		

### Hydrogen-bond geometry (Å, º)

**Table:**

*D*—H···*A*	*D*—H	H···*A*	*D*···*A*	*D*—H···*A*
O1—H1*O*1···O3^i^	0.83 (1)	1.89 (2)	2.685 (2)	159 (4)
N2—H2···O7^ii^	0.98 (1)	1.97 (2)	2.930 (3)	167 (3)
C4—H4···O2^iii^	0.95	2.31	3.204 (3)	156
C8—H8*AB*···O5^iv^	0.99	2.55	3.455 (3)	152

Symmetry codes: (i) *x*, *y*, *z*+1; (ii) −*x*+3/2, *y*+1/2, *z*−1/2; (iii) *x*−1/2, −*y*+3/2, *z*; (iv) −*x*+3/2, *y*+1/2, *z*+1/2.
